# 6-Bromo-2-(3-phenyl­allyl­idene)-2,3,4,9-tetra­hydro-1*H*-carbazol-1-one

**DOI:** 10.1107/S1600536811046563

**Published:** 2011-11-12

**Authors:** R. Velmurugan, M. Sekar, A. V. Vijayasankar, P. Ramesh, M. N. Ponnuswamy

**Affiliations:** a Post Graduate and Research Department of Chemistry, Sri Ramakrishna Mission Vidyalaya College of Arts and Science, Coimbatore 641 020, India; b Department of Engineering Chemistry, Christ University, Bangalore 560 029, Karnataka, India; c Centre of Advanced Study in Crystallography and Biophysics, University of Madras, Guindy Campus, Chennai 600 025, India

## Abstract

Mol­ecules of the title compound, C_21_H_16_BrNO, are linked through pairs of N—H⋯O inter­molecular hydrogen bonds into centrosymmetric *R*_2_^2^(10) dimers. One of the C atoms of the cyclohex-2-enone ring is disordered with refined occupancies of 0.61 (2) and 0.39 (2).

## Related literature

For the biological activity of carbazole derivatives, see: Shufen *et al.* (1995 ▶); Magnus *et al.* (1992 ▶); Abraham (1975 ▶); Saxton (1983 ▶); Phillipson & Zenk (1980 ▶); Bergman & Pelcman (1990 ▶); Bonesi *et al.* (2004 ▶); Chakraborty *et al.* (1965 ▶); Kirtikar & Basu (1933 ▶); Chakraborty *et al.* (1973 ▶); Knolker & Reddy, 2002 ▶. For puckering parameters, see: Cremer & Pople (1975 ▶). For asymmetry parameters, see: Nardelli (1983 ▶). For hydrogen-bond motifs, see: Bernstein *et al.* (1995 ▶).
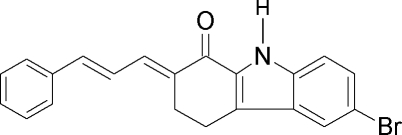


## Experimental

### 

#### Crystal data

C_21_H_16_BrNO
*M* *_r_* = 378.26
Monoclinic, 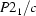

*a* = 17.3682 (7) Å
*b* = 14.9974 (8) Å
*c* = 6.6861 (3) Å
β = 92.226 (2)°
*V* = 1740.27 (14) Å^3^
*Z* = 4
Mo *K*α radiation
μ = 2.37 mm^−1^
*T* = 293 K
0.20 × 0.17 × 0.16 mm

#### Data collection

Bruker SMART APEX CCD detector diffractometer
Absorption correction: multi-scan (*SADABS*; Bruker, 1998 ▶) *T*_min_ = 0.629, *T*_max_ = 0.685
16684 measured reflections
4319 independent reflections
2158 reflections with *I* > 2σ(*I*)
*R*_int_ = 0.044

#### Refinement

*R*[*F* ^2^ > 2σ(*F* ^2^)] = 0.044
*wR*(*F* ^2^) = 0.137
*S* = 0.97
4319 reflections
231 parameters
H atoms treated by a mixture of independent and constrained refinement
Δρ_max_ = 0.46 e Å^−3^
Δρ_min_ = −0.55 e Å^−3^

### 

Data collection: *SMART* (Bruker, 1998 ▶); cell refinement: *SAINT-Plus* (Bruker, 1998 ▶); data reduction: *SAINT-Plus*; program(s) used to solve structure: *SHELXS97* (Sheldrick, 2008 ▶); program(s) used to refine structure: *SHELXL97* (Sheldrick, 2008 ▶); molecular graphics: *ORTEP-3* (Farrugia, 1997 ▶); software used to prepare material for publication: *SHELXL97* and *PLATON* (Spek, 2009 ▶).

## Supplementary Material

Crystal structure: contains datablock(s) global, I. DOI: 10.1107/S1600536811046563/bt5677sup1.cif

Structure factors: contains datablock(s) I. DOI: 10.1107/S1600536811046563/bt5677Isup2.hkl

Supplementary material file. DOI: 10.1107/S1600536811046563/bt5677Isup3.cml

Additional supplementary materials:  crystallographic information; 3D view; checkCIF report

## Figures and Tables

**Table 1:** Hydrogen-bond geometry (Å, °)

*D*—H⋯*A*	*D*—H	H⋯*A*	*D*⋯*A*	*D*—H⋯*A*
N1—H1⋯O1^i^	0.82 (3)	2.02 (3)	2.813 (3)	161 (3)

Symmetry code: (i) 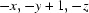.

**Fig. 1.**
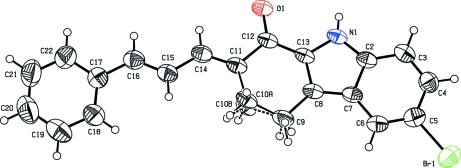
The molecular structure of the title compound, showing the atomic numbering and displacement ellipsoids drawn at the 50% probability level.

**Fig. 2.**
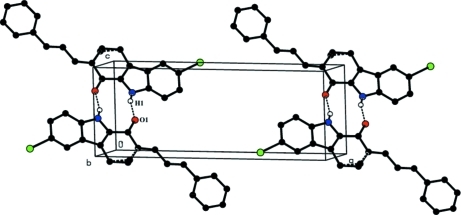
The crystal packing of the title compound. H atoms not involved in hydrogen bonding (dashed lines) have been omitted for clarity.

## supplementary crystallographic information

### Comment

Carbazole alkaloids obtained from naturally occurring sources have been the subject of extensive research, mainly because of their widespread applications in traditional medicine (Bergman & Pelcman, 1990; Bonesi *et al.*, 2004; Chakraborty *et al.*, 1965; Kirtikar & Basu, 1933). Aminocarbazoles are widely used as intermediates for the preparation of carbazole-based synthetic dyes, agrochemicals, pharmaceuticals and light-sensitive materials (Shufen *et al.*, 1995). Tetrahydrocarbazole systems are present in the framework of a number of indole-type alkaloids of biological interest (Magnus *et al*., 1992; Abraham, 1975; Saxton, 1983; Phillipson *et al.*, 1980). These types of compounds possess significant antibiotic, anti-carcinogenic, antiviral and anti-inflammatory properties (Chakraborty *et al.*, 1973). The chemists have been attracted towards these compounds due to their biological activities and potential applications as pharmacological agents (Knolker & Reddy, 2002). Against this background and to ascertain the molecular structure and conformation, the X-ray crystal structure determination of the title compound has been carried out.

The *ORTEP* plot of the molecule is shown in Fig. 1. One of the C atoms of the cyclohexane ring is disordered with refined occupancies of 0.61 (2) and 0.39 (2). The disordered position of C10B in the cyclohexane ring in the carbazole ring system adopts envelope conformation with the puckering parameters (Cremer & Pople, 1975) and the asymmetry parameters (Nardelli, 1983) are: q_2_=0.242 (7) Å, q_3_ = 0.171 (6) Å, φ_2_ = 117.3 (13)° and Δ_s_(C10B & C13)= 1.5 (5)°. The sum of the bond angles around N1 [358.3°] is in accordance with *sp^2^* hybridization.

The crystal packing reveals that symmetry-related molecules are linked through N—H···O  intermolecular hydrogen bonds into cyclic centrosymmetric *R*_2_^2^(10) dimers.

### Experimental

The mixed aldol condensation reaction of 6-bromo-1-oxo-1,2,3, 4-tetrahydrocarbazole reacted with cinnamaldehyde in the presence of alcoholic KOH, afforded a single product, substituted 6-Bromo-2-(3-phenyl-allylidene) -2,3,4,9-tetrahydro-carbazol-1-one. This was purified by using column chromatography over silica gel (mesh 60–80). During elution of the column with petroleum ether (60–80°C) and ethyl acetate [1:2] mixture, a yellowish solid was obtained. It was recrystallized from the solvent mixture ethyl acetate and acetone (8:2).

### Refinement

The N-bound H atom was located in a difference map and refined isotropically. C-bound H atoms were positioned geometrically (C–H = 0.93–0.97 Å) and allowed to ride on their parent atoms, with *U*_iso_(H) = 1.2*U*_eq_(C) for all H atoms.

### Crystal data

**Table:**

C_21_H_16_BrNO	*F*(000) = 768
*M**_r_* = 378.26	*D*_x_ = 1.444 Mg m^−^^3^
Monoclinic, *P*2_1_/*c*	Mo *K*α radiation, λ = 0.71073 Å
Hall symbol: -P 2ybc	Cell parameters from 2134 reflections
*a* = 17.3682 (7) Å	θ = 1.2–28.3°
*b* = 14.9974 (8) Å	µ = 2.37 mm^−^^1^
*c* = 6.6861 (3) Å	*T* = 293 K
β = 92.226 (2)°	Block, yellow
*V* = 1740.27 (14) Å^3^	0.20 × 0.17 × 0.16 mm
*Z* = 4	

### Data collection

**Table:**

Bruker SMART APEX CCD detector diffractometer	4319 independent reflections
Radiation source: fine-focus sealed tube	2158 reflections with *I* > 2σ(*I*)
graphite	*R*_int_ = 0.044
ω scans	θ_max_ = 28.3°, θ_min_ = 1.2°
Absorption correction: multi-scan (*SADABS*; Bruker, 1998)	*h* = −23→23
*T*_min_ = 0.629, *T*_max_ = 0.685	*k* = −18→20
16684 measured reflections	*l* = −8→8

### Refinement

**Table:**

Refinement on *F*^2^	Primary atom site location: structure-invariant direct methods
Least-squares matrix: full	Secondary atom site location: difference Fourier map
*R*[*F*^2^ > 2σ(*F*^2^)] = 0.044	Hydrogen site location: inferred from neighbouring sites
*wR*(*F*^2^) = 0.137	H atoms treated by a mixture of independent and constrained refinement
*S* = 0.97	*w* = 1/[σ^2^(*F*_o_^2^) + (0.0592*P*)^2^ + 0.6068*P*] where *P* = (*F*_o_^2^ + 2*F*_c_^2^)/3
4319 reflections	(Δ/σ)_max_ < 0.001
231 parameters	Δρ_max_ = 0.46 e Å^−^^3^
0 restraints	Δρ_min_ = −0.55 e Å^−^^3^

### Special details

**Table:**

Geometry. All e.s.d.'s (except the e.s.d. in the dihedral angle between two l.s. planes) are estimated using the full covariance matrix. The cell e.s.d.'s are taken into account individually in the estimation of e.s.d.'s in distances, angles and torsion angles; correlations between e.s.d.'s in cell parameters are only used when they are defined by crystal symmetry. An approximate (isotropic) treatment of cell e.s.d.'s is used for estimating e.s.d.'s involving l.s. planes.
Refinement. Refinement of *F*^2^ against ALL reflections. The weighted *R*-factor *wR* and goodness of fit *S* are based on *F*^2^, conventional *R*-factors *R* are based on *F*, with *F* set to zero for negative *F*^2^. The threshold expression of *F*^2^ > σ(*F*^2^) is used only for calculating *R*-factors(gt) *etc*. and is not relevant to the choice of reflections for refinement. *R*-factors based on *F*^2^ are statistically about twice as large as those based on *F*, and *R*- factors based on ALL data will be even larger.

### Fractional atomic coordinates and isotropic or equivalent isotropic displacement parameters (Å^2^)

**Table:**

	*x*	*y*	*z*	*U*_iso_*/*U*_eq_	Occ. (<1)
Br1	0.39020 (2)	0.32240 (3)	0.51370 (6)	0.1026 (2)	
O1	−0.08065 (12)	0.45133 (15)	0.1631 (3)	0.0688 (6)	
N1	0.08292 (14)	0.42507 (17)	0.1535 (3)	0.0552 (6)	
H1	0.0713 (17)	0.458 (2)	0.059 (4)	0.067 (9)*	
C2	0.15712 (17)	0.40282 (18)	0.2075 (4)	0.0528 (7)	
C3	0.22410 (18)	0.4135 (2)	0.1039 (4)	0.0641 (8)	
H3	0.2229	0.4378	−0.0242	0.077*	
C4	0.29216 (19)	0.3873 (2)	0.1963 (5)	0.0675 (8)	
H4	0.3381	0.3937	0.1306	0.081*	
C5	0.2929 (2)	0.3509 (2)	0.3901 (5)	0.0692 (9)	
C6	0.22789 (19)	0.3375 (2)	0.4921 (4)	0.0622 (8)	
H6	0.2299	0.3118	0.6187	0.075*	
C7	0.15750 (17)	0.36386 (18)	0.4003 (4)	0.0505 (7)	
C8	0.08023 (17)	0.36309 (18)	0.4592 (3)	0.0485 (6)	
C9	0.04470 (19)	0.3290 (2)	0.6440 (4)	0.0658 (8)	
H9A	0.0487	0.2645	0.6453	0.079*	0.61 (2)
H9B	0.0743	0.3513	0.7595	0.079*	0.61 (2)
H9C	0.0547	0.3712	0.7519	0.079*	0.39 (2)
H9D	0.0691	0.2730	0.6823	0.079*	0.39 (2)
C11	−0.08446 (18)	0.38062 (19)	0.4825 (4)	0.0538 (7)	
C12	−0.04532 (17)	0.41447 (18)	0.3044 (3)	0.0506 (7)	
C13	0.03659 (16)	0.40161 (17)	0.3067 (3)	0.0477 (6)	
C14	−0.15949 (18)	0.3978 (2)	0.4961 (4)	0.0586 (7)	
H14	−0.1826	0.4283	0.3886	0.070*	
C15	−0.20892 (18)	0.3753 (2)	0.6550 (4)	0.0631 (8)	
H15	−0.1887	0.3416	0.7613	0.076*	
C16	−0.28207 (19)	0.4001 (2)	0.6583 (4)	0.0676 (8)	
H16	−0.3011	0.4311	0.5464	0.081*	
C17	−0.33642 (18)	0.3850 (2)	0.8155 (5)	0.0658 (8)	
C18	−0.3143 (2)	0.3524 (2)	1.0019 (5)	0.0780 (10)	
H18	−0.2625	0.3403	1.0313	0.094*	
C19	−0.3677 (3)	0.3376 (3)	1.1451 (6)	0.0984 (13)	
H19	−0.3521	0.3145	1.2692	0.118*	
C20	−0.4435 (3)	0.3568 (3)	1.1050 (8)	0.1123 (16)	
H20	−0.4795	0.3470	1.2019	0.135*	
C21	−0.4661 (2)	0.3902 (4)	0.9231 (8)	0.1155 (16)	
H21	−0.5178	0.4033	0.8962	0.139*	
C22	−0.4137 (2)	0.4048 (3)	0.7794 (6)	0.0882 (11)	
H22	−0.4300	0.4282	0.6561	0.106*	
C10A	−0.0342 (11)	0.3529 (15)	0.665 (2)	0.058 (4)	0.39 (2)
H10A	−0.0353	0.4016	0.7602	0.069*	0.39 (2)
H10B	−0.0594	0.3026	0.7258	0.069*	0.39 (2)
C10B	−0.0401 (7)	0.3148 (9)	0.6188 (17)	0.059 (2)	0.61 (2)
H10C	−0.0619	0.3169	0.7501	0.071*	0.61 (2)
H10D	−0.0487	0.2551	0.5663	0.071*	0.61 (2)

### Atomic displacement parameters (Å^2^)

**Table:**

	*U*^11^	*U*^22^	*U*^33^	*U*^12^	*U*^13^	*U*^23^
Br1	0.0700 (3)	0.1349 (5)	0.1014 (3)	−0.0016 (2)	−0.0135 (2)	0.0210 (2)
O1	0.0752 (13)	0.0818 (16)	0.0491 (10)	0.0044 (11)	−0.0026 (9)	0.0253 (10)
N1	0.0740 (16)	0.0542 (16)	0.0373 (11)	0.0027 (13)	0.0008 (11)	0.0120 (11)
C2	0.0742 (19)	0.0405 (16)	0.0434 (13)	0.0007 (14)	−0.0004 (13)	0.0012 (12)
C3	0.082 (2)	0.060 (2)	0.0505 (15)	−0.0032 (17)	0.0076 (15)	0.0044 (14)
C4	0.071 (2)	0.064 (2)	0.0688 (19)	−0.0054 (17)	0.0094 (15)	0.0014 (16)
C5	0.073 (2)	0.064 (2)	0.070 (2)	−0.0028 (16)	−0.0075 (17)	0.0020 (16)
C6	0.074 (2)	0.062 (2)	0.0499 (15)	−0.0001 (16)	−0.0078 (14)	0.0063 (14)
C7	0.0710 (18)	0.0384 (16)	0.0417 (13)	−0.0026 (13)	−0.0026 (12)	0.0030 (11)
C8	0.0729 (18)	0.0351 (15)	0.0372 (12)	−0.0008 (13)	−0.0034 (12)	−0.0007 (11)
C9	0.082 (2)	0.071 (2)	0.0438 (15)	−0.0037 (18)	−0.0009 (14)	0.0188 (14)
C11	0.0754 (19)	0.0448 (17)	0.0409 (13)	0.0034 (15)	0.0012 (12)	0.0033 (12)
C12	0.0739 (18)	0.0415 (16)	0.0363 (12)	0.0008 (14)	0.0002 (12)	0.0021 (11)
C13	0.0715 (18)	0.0374 (15)	0.0343 (12)	−0.0006 (13)	0.0028 (12)	0.0011 (11)
C14	0.075 (2)	0.0493 (18)	0.0510 (15)	0.0002 (15)	−0.0005 (14)	0.0049 (13)
C15	0.074 (2)	0.061 (2)	0.0544 (16)	−0.0034 (16)	0.0019 (14)	0.0079 (14)
C16	0.076 (2)	0.064 (2)	0.0621 (18)	−0.0054 (17)	−0.0012 (15)	0.0070 (15)
C17	0.069 (2)	0.056 (2)	0.0716 (19)	−0.0089 (16)	0.0036 (16)	−0.0021 (16)
C18	0.086 (2)	0.075 (2)	0.074 (2)	−0.0008 (19)	0.0086 (18)	0.0047 (18)
C19	0.132 (4)	0.091 (3)	0.074 (2)	−0.016 (3)	0.019 (3)	0.006 (2)
C20	0.105 (4)	0.130 (4)	0.105 (3)	−0.033 (3)	0.037 (3)	−0.021 (3)
C21	0.070 (2)	0.158 (5)	0.119 (4)	−0.018 (3)	0.014 (3)	−0.026 (3)
C22	0.073 (2)	0.100 (3)	0.091 (2)	−0.006 (2)	−0.001 (2)	−0.005 (2)
C10A	0.084 (7)	0.049 (10)	0.041 (6)	0.023 (8)	0.017 (5)	0.013 (5)
C10B	0.088 (4)	0.048 (6)	0.042 (4)	0.006 (5)	0.009 (3)	0.010 (3)

### Geometric parameters (Å, °)

**Table:**

Br1—C5	1.900 (3)	C11—C10B	1.532 (11)
O1—C12	1.237 (3)	C11—C10A	1.529 (16)
N1—C2	1.366 (4)	C12—C13	1.435 (4)
N1—C13	1.373 (3)	C14—C15	1.432 (4)
N1—H1	0.82 (3)	C14—H14	0.9300
C2—C3	1.386 (4)	C15—C16	1.325 (4)
C2—C7	1.415 (4)	C15—H15	0.9300
C3—C4	1.370 (4)	C16—C17	1.458 (4)
C3—H3	0.9300	C16—H16	0.9300
C4—C5	1.406 (4)	C17—C18	1.379 (4)
C4—H4	0.9300	C17—C22	1.387 (4)
C5—C6	1.357 (5)	C18—C19	1.377 (5)
C6—C7	1.403 (4)	C18—H18	0.9300
C6—H6	0.9300	C19—C20	1.363 (6)
C7—C8	1.413 (4)	C19—H19	0.9300
C8—C13	1.374 (4)	C20—C21	1.359 (6)
C8—C9	1.492 (4)	C20—H20	0.9300
C9—C10A	1.429 (17)	C21—C22	1.366 (5)
C9—C10B	1.491 (12)	C21—H21	0.9300
C9—H9A	0.9700	C22—H22	0.9300
C9—H9B	0.9700	C10A—H10A	0.9700
C9—H9C	0.9700	C10A—H10B	0.9700
C9—H9D	0.9700	C10B—H10C	0.9700
C11—C14	1.335 (4)	C10B—H10D	0.9700
C11—C12	1.483 (4)		
			
C2—N1—C13	108.3 (2)	C14—C11—C10A	121.7 (6)
C2—N1—H1	123 (2)	C12—C11—C10A	117.9 (7)
C13—N1—H1	127 (2)	C10B—C11—C10A	24.7 (5)
N1—C2—C3	129.9 (2)	O1—C12—C13	122.0 (2)
N1—C2—C7	108.2 (2)	O1—C12—C11	122.5 (3)
C3—C2—C7	121.9 (3)	C13—C12—C11	115.5 (2)
C4—C3—C2	117.9 (3)	N1—C13—C8	109.8 (2)
C4—C3—H3	121.1	N1—C13—C12	124.6 (2)
C2—C3—H3	121.1	C8—C13—C12	125.6 (2)
C3—C4—C5	120.3 (3)	C11—C14—C15	128.3 (3)
C3—C4—H4	119.9	C11—C14—H14	115.8
C5—C4—H4	119.9	C15—C14—H14	115.8
C6—C5—C4	122.8 (3)	C16—C15—C14	123.3 (3)
C6—C5—Br1	119.5 (2)	C16—C15—H15	118.3
C4—C5—Br1	117.7 (3)	C14—C15—H15	118.3
C5—C6—C7	117.9 (3)	C15—C16—C17	128.1 (3)
C5—C6—H6	121.1	C15—C16—H16	116.0
C7—C6—H6	121.1	C17—C16—H16	116.0
C6—C7—C8	134.2 (2)	C18—C17—C22	117.9 (3)
C6—C7—C2	119.2 (3)	C18—C17—C16	122.7 (3)
C8—C7—C2	106.6 (2)	C22—C17—C16	119.4 (3)
C13—C8—C7	107.0 (2)	C19—C18—C17	120.9 (4)
C13—C8—C9	121.6 (3)	C19—C18—H18	119.6
C7—C8—C9	131.4 (2)	C17—C18—H18	119.6
C10A—C9—C8	115.1 (6)	C20—C19—C18	120.1 (4)
C10A—C9—C10B	25.8 (6)	C20—C19—H19	120.0
C8—C9—C10B	113.2 (4)	C18—C19—H19	120.0
C10A—C9—H9A	108.5	C21—C20—C19	119.8 (4)
C8—C9—H9A	108.5	C21—C20—H20	120.1
C10B—C9—H9A	85.8	C19—C20—H20	120.1
C10A—C9—H9B	108.5	C20—C21—C22	120.7 (4)
C8—C9—H9B	108.5	C20—C21—H21	119.6
C10B—C9—H9B	129.1	C22—C21—H21	119.6
H9A—C9—H9B	107.5	C21—C22—C17	120.7 (4)
C10A—C9—H9C	84.7	C21—C22—H22	119.7
C8—C9—H9C	108.9	C17—C22—H22	119.7
C10B—C9—H9C	108.9	C9—C10A—C11	120.7 (9)
H9A—C9—H9C	129.3	C9—C10A—H10A	107.2
H9B—C9—H9C	27.1	C11—C10A—H10A	107.2
C10A—C9—H9D	127.0	C9—C10A—H10B	107.2
C8—C9—H9D	108.9	C11—C10A—H10B	107.2
C10B—C9—H9D	108.9	H10A—C10A—H10B	106.8
H9A—C9—H9D	26.4	C9—C10B—C11	116.5 (7)
H9B—C9—H9D	82.8	C9—C10B—H10C	108.2
H9C—C9—H9D	107.8	C11—C10B—H10C	108.2
C14—C11—C12	117.9 (2)	C9—C10B—H10D	108.2
C14—C11—C10B	123.6 (5)	C11—C10B—H10D	108.2
C12—C11—C10B	117.6 (5)	H10C—C10B—H10D	107.3
			
C13—N1—C2—C3	−179.7 (3)	C9—C8—C13—N1	−178.6 (3)
C13—N1—C2—C7	0.2 (3)	C7—C8—C13—C12	−179.9 (3)
N1—C2—C3—C4	178.2 (3)	C9—C8—C13—C12	0.5 (4)
C7—C2—C3—C4	−1.8 (4)	O1—C12—C13—N1	−2.5 (4)
C2—C3—C4—C5	0.0 (5)	C11—C12—C13—N1	177.5 (2)
C3—C4—C5—C6	1.8 (5)	O1—C12—C13—C8	178.6 (3)
C3—C4—C5—Br1	−176.5 (2)	C11—C12—C13—C8	−1.5 (4)
C4—C5—C6—C7	−1.8 (5)	C12—C11—C14—C15	−177.5 (3)
Br1—C5—C6—C7	176.6 (2)	C10B—C11—C14—C15	13.7 (8)
C5—C6—C7—C8	−178.1 (3)	C10A—C11—C14—C15	−15.7 (12)
C5—C6—C7—C2	0.0 (4)	C11—C14—C15—C16	176.1 (3)
N1—C2—C7—C6	−178.1 (3)	C14—C15—C16—C17	−176.8 (3)
C3—C2—C7—C6	1.8 (4)	C15—C16—C17—C18	10.0 (6)
N1—C2—C7—C8	0.4 (3)	C15—C16—C17—C22	−170.7 (4)
C3—C2—C7—C8	−179.6 (3)	C22—C17—C18—C19	1.9 (5)
C6—C7—C8—C13	177.4 (3)	C16—C17—C18—C19	−178.8 (3)
C2—C7—C8—C13	−0.9 (3)	C17—C18—C19—C20	−1.3 (6)
C6—C7—C8—C9	−3.1 (5)	C18—C19—C20—C21	0.3 (7)
C2—C7—C8—C9	178.7 (3)	C19—C20—C21—C22	0.1 (8)
C13—C8—C9—C10A	−10.2 (12)	C20—C21—C22—C17	0.6 (7)
C7—C8—C9—C10A	170.3 (12)	C18—C17—C22—C21	−1.5 (6)
C13—C8—C9—C10B	18.1 (7)	C16—C17—C22—C21	179.2 (4)
C7—C8—C9—C10B	−161.4 (7)	C8—C9—C10A—C11	21 (2)
C14—C11—C12—O1	−5.8 (4)	C10B—C9—C10A—C11	−71 (2)
C10B—C11—C12—O1	163.7 (6)	C14—C11—C10A—C9	175.7 (12)
C10A—C11—C12—O1	−168.3 (10)	C12—C11—C10A—C9	−22 (2)
C14—C11—C12—C13	174.2 (3)	C10B—C11—C10A—C9	73.4 (19)
C10B—C11—C12—C13	−16.3 (7)	C10A—C9—C10B—C11	65.1 (18)
C10A—C11—C12—C13	11.7 (11)	C8—C9—C10B—C11	−34.9 (12)
C2—N1—C13—C8	−0.8 (3)	C14—C11—C10B—C9	−155.8 (6)
C2—N1—C13—C12	−179.8 (3)	C12—C11—C10B—C9	35.4 (12)
C7—C8—C13—N1	1.0 (3)	C10A—C11—C10B—C9	−62 (2)

### Hydrogen-bond geometry (Å, °)

**Table:**

*D*—H···*A*	*D*—H	H···*A*	*D*···*A*	*D*—H···*A*
N1—H1···O1^i^	0.82 (3)	2.02 (3)	2.813 (3)	161 (3)

Symmetry codes: (i) −*x*, −*y*+1, −*z*.
